# Case Report: Sturge-Weber syndrome with contralateral morning glory syndrome

**DOI:** 10.3389/fmed.2026.1773788

**Published:** 2026-03-16

**Authors:** Liang Wang, Manhong Li, Guorui Dou, Liying Zheng, Ziyi Zhou, Jiaxing Sun, Yusheng Wang, Zifeng Zhang

**Affiliations:** 1 Department of Ophthalmology, Eye Institute of Chinese PLA, Xijing Hospital, Fourth Military Medical University, Xi’an, China; 2 The Ministry of Education Key Lab of Hazard Assessment and Control in Special Operational Environment, School of Public Health, Fourth Military Medical University, Xi’an, China

**Keywords:** management, morning glory syndrome, multimodal imaging, pathogenesis, Sturge-Weber syndrome

## Abstract

**Background:**

Sturge-Weber syndrome (SWS) and morning glory syndrome (MGS) are rare congenital disorders that present significant diagnostic challenges. This case describes a girl whose ocular examination revealed distinct manifestations of these two rare but etiologically separate congenital anomalies, a combination that has never been reported before.

**Case presentation:**

A 9-year-old girl came to our ophthalmology department complaining of blurred vision in her left eye that had lasted for more than 6 months. On examination, a port-wine birthmark was confirmed on the right facial side. The visual acuity was normal in the right eye (20/20) but severely reduced in the left, improving only from counting fingers to 20/1000 with correction. Funduscopy revealed diffuse choroidal hemangiomas in the right eye and an enlarged optic disc with a central funnel-shaped excavation and radiating vascular anomalies accompanied by retinal detachment in the left eye. The patient was ultimately diagnosed with SWS affecting the right eye and MGS with retinal detachment in the left eye.

**Conclusion:**

This case is noteworthy as it documents the first instance of SWS and MGS coexisting in one patient, supported by imaging findings, thereby challenging established paradigms regarding their individual clinical manifestations and underlying pathogenesis. For patients with port-wine birthmark or unilateral optic disc anomalies, thorough assessment of the contralateral eye and comprehensive neurological evaluation are essential to detect associated conditions that may threaten vision and cause neurological damage.

## 1 Introduction

The Sturge-Weber syndrome (SWS) is a rare sporadic congenital neurocutaneous disorder, characterized by facial and leptomeningeal capillary malformations, ocular vascular malformations, and may be accompanied by many other manifestations (1). The predominant ocular manifestations associated with SWS include glaucoma and choroidal hemangioma. Glaucoma may manifest during infancy, or it may develop later in life (2–4). Additionally, neurological complications are frequently observed in individuals with SWS, affecting approximately 70 to 80% of patients, with seizures being the most prevalent complication (3, 5–7). Morning glory syndrome (MGS) is a rare congenital optic disc anomaly characterized by an excavated disc that resembles a blooming morning glory flower (8). It is always accompanied by peripapillary pigmentation, a radiating pattern of retinal blood vessels, and a central white tuft of glial tissue (9). MGS is observed to be more prevalent in females, with a ratio of 2:1 when compared to males (10). Typically, it manifests unilaterally and presents in early childhood. This syndrome is associated with severely compromised visual function and prognosis, generally resulting in a visual acuity of less than 0.1 decimal visual acuity (10). The initial clinical presentation of MGS may manifest in several ways, including reduced visual acuity associated with posterior segment involvement, microphthalmia, retinal detachment, leukocoria, and, although less frequently observed, strabismus (9, 11–13). Diagnosis is primarily clinical and is further corroborated by retinal imaging techniques (9).

This case describes a girl whose ocular examination revealed distinct manifestations of these two rare but etiologically separate congenital anomalies. A comprehensive literature search was conducted in PubMed and Web of Science. The search confirmed that, although isolated cases of SWS or MGS have been reported and a few reports have documented SWS associated with other ocular anomalies, no previous publication has described the coexistence of SWS and MGS in the same patient with contralateral involvement.

## 2 Case report

A 9-year-old girl came to our ophthalmology department complaining of blurred vision in her left eye that had lasted for more than 6 months. The patient had a port-wine birthmark on the right side of her face, diagnosed at age 2 in the dermatology department of another hospital. Although ophthalmologic referral was recommended then, further evaluation was not pursued because the child did not comply.

**Figure 1:**
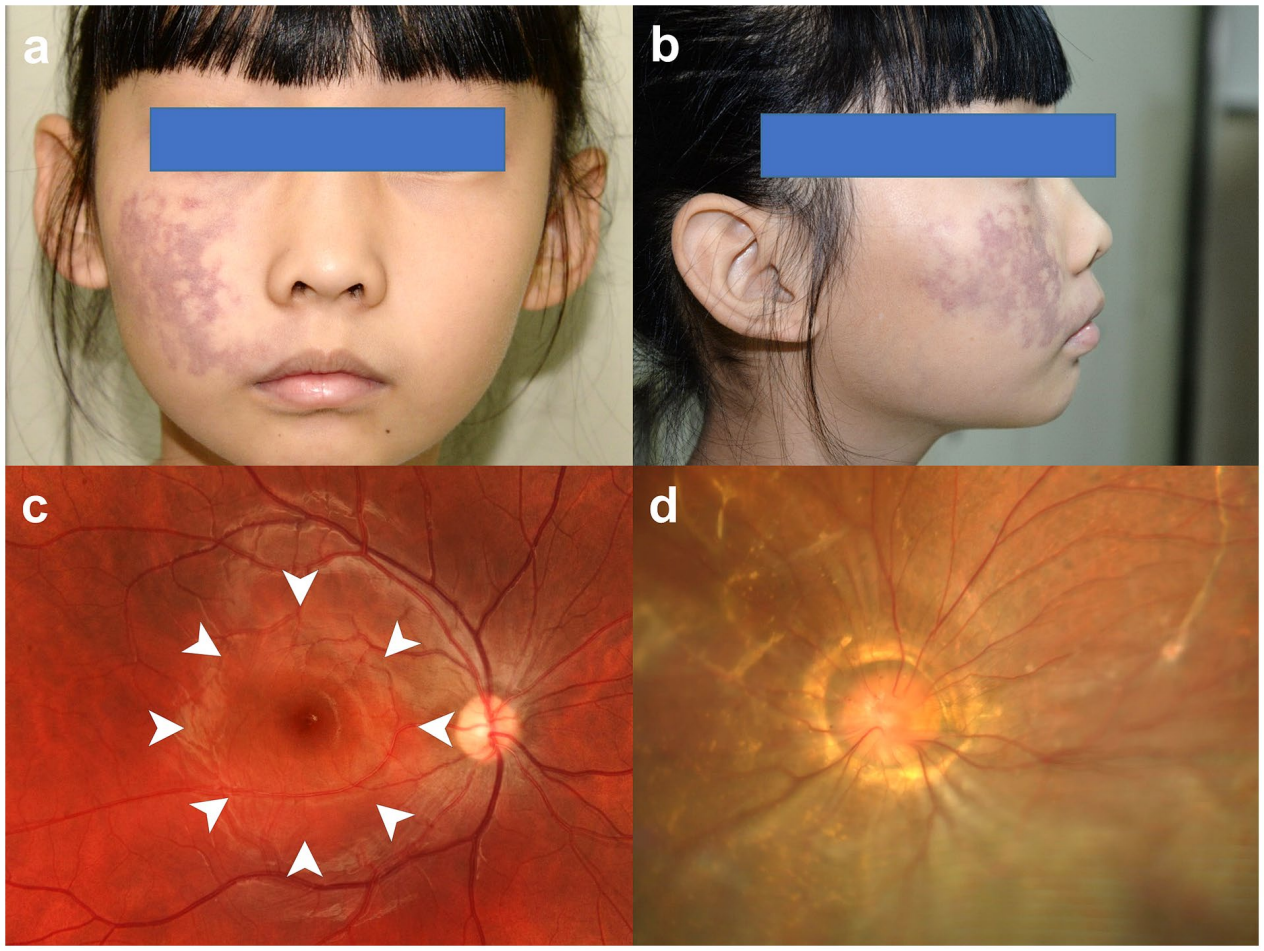
External appearance and fundus photographs at the time of examination. **(a,b)** Frontal and lateral photographs show that the port-wine birthmark was treated with laser therapy at the age 2. **(c)** Fundus photograph of the right eye shows diffuse choroidal hemangiomas temporal to the optic disc, which appear as focal orange-red changes (arrowheads). **(d)** Fundus photograph of the left eye shows an enlarged optic disc with a central funnel-shaped excavation and radiating vascular anomalies. The presence of a bluish-gray elevated retina with subretinal fibrous strands in the left eye indicates a chronic exudative retinal detachment.

On examination, a port-wine birthmark was confirmed on the right facial side (Figures 1a,b). The patient had never experienced any type of seizure, including febrile seizures, focal seizures, or generalized convulsions. No antiepileptic medications had ever been prescribed. She had no history of hemiparesis, focal motor deficits, transient stroke-like episodes, or unexplained headaches. Her developmental milestones had been age-appropriate and she currently attends regular school and performs adequately for her age. Formal neurological examination revealed no focal neurological signs, and brain magnetic resonance angiography was unremarkable. A three-generation family pedigree was obtained. The patient was an only child, whose parents were non-consanguineous and both healthy. No family members had a history of port-wine stains, seizures, glaucoma, early-onset stroke, or any known neurocutaneous syndromes such as SWS and tuberous sclerosis.

**Figure 2:**
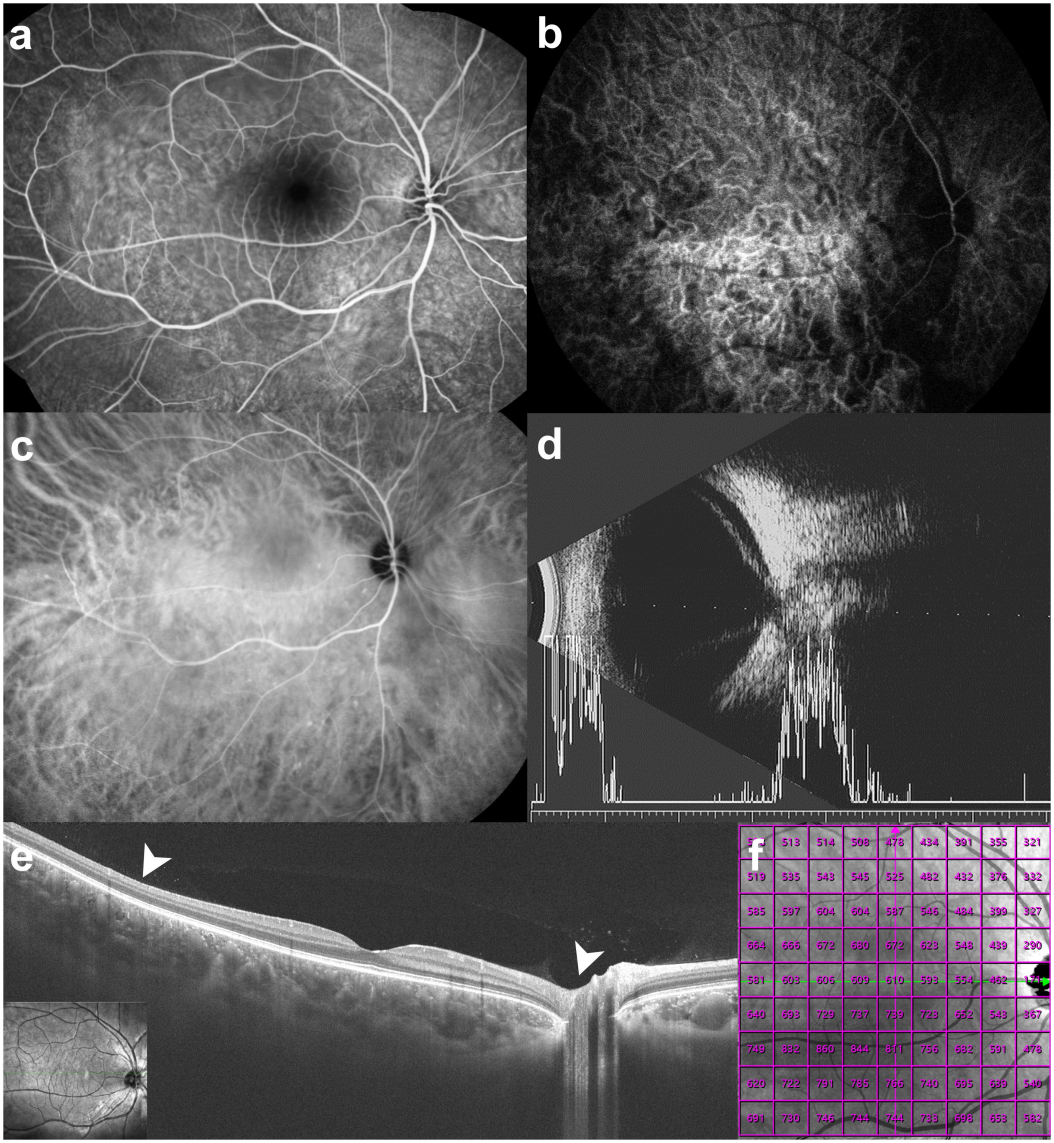
Multimodal imaging examination images of the eyes. **(a)** Fluorescein fundus angiography of the right eye shows diffuse hyperfluorescence in the choroidal hemangioma area (at 6 min and 4 s after injection). **(b)** Indocyanine green angiography shows immediate filling of the choroidal hemangioma at 1 s after injection. **(c)** The lesion displays a coarse, “spongy” vascular pattern with well-demarcated, homogeneous hyperfluorescence at 47 s after injection. **(d)** B-scan ultrasonography of the left eye reveals a funnel-shaped excavation of the posterior fundus centered on the optic disc, with an irregular, hypoechoic area within it. A V-shaped band of moderate to low echogenicity surrounds the disc, which is highly suggestive of exudative retinal detachment. **(e)** Optical coherence tomography reveals marked thickening of the choroid with homogeneous, low-reflective luminal spaces. **(f)** Quantitative analysis confirms a significant increase in choroidal thickness in the area indicated by the two arrowheads in panel **e**.

The patient had no prior history of eye trauma and surgery. The visual acuity was normal in the right eye (20/20) but severely reduced in the left, improving only from counting fingers to 20/1000 with correction. Intraocular pressure was within normal range bilaterally. The anterior segment examination of the right eye was unremarkable. Funduscopy revealed diffuse choroidal hemangiomas temporal to the optic disc. The lesion measured 4.1 disc diameters (DD) in its greatest horizontal diameter and 4.2 DD in its greatest vertical diameter, appearing as focal orange-red changes (Figure 1c). Funduscopy of the left eye showed an enlarged optic disc with a central funnel-shaped excavation and radiating vascular anomalies. Exudative retinal detachment involving the posterior pole was observed, with no identifiable retinal breaks or tears on thorough examination (Figure 1d). Fluorescein fundus angiography of the right eye showed diffuse hyperfluorescence (Figure 2a). Indocyanine green angiography showed immediate filling of the choroidal hemangioma and the lesion displayed a coarse, “spongy” vascular pattern with well-demarcated, homogeneous hyperfluorescence (Figures 2b,c). B-scan ultrasonography of the left eye revealed a funnel-shaped excavation of the posterior fundus centered on the optic disc. The excavation measured 4.3 mm in transverse diameter and 3.4 mm in depth. No echogenic band suggestive of retinal break was identified. Extensive exudative retinal detachment involving the posterior pole was observed. These ultrasonographic findings are characteristic of MGS and correlate with the funduscopic findings (Figure 2d). Optical coherence tomography revealed marked thickening of the choroid with homogeneous, low-reflective luminal spaces, which are consistent with the underlying diffuse choroidal hemangioma typical of SWS (Figures 2e,f). The diagnosis of SWS relies on distinct criteria, affirming the diagnosis when a minimum of two of the following three conditions are satisfied: the presence of a distinctive facial port-wine birthmark, ocular vascular anomalies, and particular neuroimaging results that reveal leptomeningeal capillary malformations (14). The patient was ultimately diagnosed with SWS affecting the right eye and MGS with retinal detachment in the left eye.

Surgical intervention for the retinal detachment was deemed to offer a low probability of anatomic and functional benefit. Coupled with the wishes of the patient’s family, a conservative treatment plan consisting of regular clinical observation without surgical intervention was ultimately adopted. The patient was scheduled for periodic ophthalmologic follow-up, including funduscopy, optical coherence tomography, and B-scan ultrasonography every 3 to 6 months to monitor disease progression. At 6 months, intraocular pressure was normal bilaterally. Funduscopic and B-scan findings remained stable, with no progression of retinal detachment or optic disc excavation.

## 3 Discussion

The reported incidence of SWS is estimated to be between 1 in 20,000 and 1 in 50,000 live births, whereas that of MGS is approximately 3.6 per 100,000 individuals (9, 15). As confirmed by our literature review, this represents the first reported case of contralateral SWS and MGS. Although the neurologic and ocular manifestations of SWS typically follow an ipsilateral pattern relative to the facial hemangioma (1, 16), the occurrence of a contralateral MGS is statistically highly improbable. The *R183Q* somatic nonsynonymous mosaic mutation in *GNAQ* is the predominant cause of SWS. This mutation leads to overactivation of various downstream signaling pathways, including the mitogen-activated protein kinase pathway, resulting in neurovascular abnormalities (1). Recent evidence has also linked the condition to mutations in *GNA11* and *GNB2* (16).

The pathogenesis of MGS remains unclear but may be related to abnormal development of the mesoderm and incomplete closure of the blastopore (8). Currently, there are reports suggesting that mutations in *PAX6* may be associated with MGS (8). Although SWS and MGS co-occurred in this patient, no common pathogenic mechanism has yet been identified. Whether this association is coincidental or reflects a shared developmental pathway warrants further investigation.

In addition to glaucoma and choroidal hemangioma, SWS may involve various ocular vascular malformations affecting the retina, conjunctiva, or sclera (2, 17). This diversity underscores the necessity of comprehensive bilateral ocular examination in all patients with SWS. She et al. highlighted the significant presence of nonperfused areas in the peripheral retina of children diagnosed with MGS and called for increased attention to this issue (18). Approximately 45% of individuals identified with MGS exhibit concurrent cerebrovascular abnormalities (19), which include Moyamoya disease, basal encephalocele, hypopituitarism, and midline cranial malformations (20). In addition, Poillon et al. (21) reported a series of cases, who exhibited enlargement of the optic pathways. This finding may indicate a malformation linked to MGS. Consequently, it is imperative that individuals identified with MGS receive neuroimaging assessments (21).

Although our patient’s initial brain magnetic resonance angiography imaging was unremarkable, this finding is not uncommon, as a substantial proportion of MGS patients may lack detectable vascular anomalies in the early stage. This case highlights the clinical importance of ongoing neurological surveillance and repeat imaging in MGS, even when initial studies are unremarkable, as vascular changes may develop over time.

The diagnosis of SWS in the right eye was established based on the clinical diagnostic criteria: the presence of a facial port-wine birthmark and ipsilateral diffuse choroidal hemangioma, despite unremarkable neuroimaging (14). The differential diagnosis for MGS primarily includes optic disc coloboma and persistent fetal vasculature. Unlike MGS, optic disc coloboma typically presents as a well-demarcated, bowl-shaped excavation often accompanied by inferior uveal coloboma (9). Persistent fetal vasculature is usually associated with microphthalmia, a retrolental fibrovascular membrane, and a patent hyaloid artery, none of which were observed in this patient (9).

This is a complex disorder that needs a multidisciplinary approach for optimal management (16). Surgical intervention for the retinal detachment was deemed to offer a low probability of anatomic and functional benefit. Coupled with the wishes of the patient’s family, a conservative treatment plan was ultimately adopted. At present, there is a lack of agreement regarding the optimal management strategies for choroidal hemangioma (22). The therapeutic approach is influenced by various factors, including whether the lesion is diffuse or circumscribed, visual acuity, and the need for concurrent glaucoma intervention (5–7). Glaucoma is the most common ocular complication of SWS, while 40% of patients with glaucoma have late-onset disease (16). The patient remains under close follow-up for monitoring of potential complications including glaucoma and epileptic seizures.

## 4 Conclusion

This case is noteworthy as it documents the first instance of SWS and MGS coexisting in one patient, supported by imaging findings, thereby challenging established paradigms regarding their individual clinical manifestations and underlying pathogenesis. This unique presentation raises questions about potential shared pathogenic mechanisms. More documentation of similar cases in the future will definitively classify this finding and refine our understanding of the underlying pathophysiology of SWS and MGS. In addition, for patients with port-wine birthmark or unilateral optic disc anomalies, thorough assessment of the contralateral eye and comprehensive neurological evaluation are essential to detect associated conditions that may threaten vision and cause neurological damage.

## Data Availability

The original contributions presented in the study are included in the article/supplementary material, further inquiries can be directed to the corresponding authors.
